# Frequency of pain and eating disorders among professional and amateur dancers: Frequência de dor e distúrbios alimentares entre bailarinos profissionais e amadores

**DOI:** 10.1590/1516-3180.2016.0077310516

**Published:** 2016-09-26

**Authors:** Maria Angélica Kurpel Diogo, Gabriel Gomes de Oliveira Ribas, Thelma Larocca Skare

**Affiliations:** I BSc. Medical Student, Faculdade Evangélica do Paraná (FEPAR), Curitiba, PR, Brazil.; II MD, PhD. Head of Rheumatology Unit, Hospital Universitário Evangélico de Curitiba, Curitiba, PR, Brazil.

**Keywords:** Musculoskeletal pain, Feeding and eating disorders, Bulimia, Anxiety, Dancing, Dor musculoesquelética, Transtornos da alimentação e da ingestão de alimentos, Bulimia, Ansiedade, Dança

## Abstract

**CONTEXT AND OBJECTIVE:**

The pursuit of perfection can cause anxiety and lead dancers to exceed their physical limits. The aim here was to evaluate the prevalence of pain symptoms and eating disorders among professional and amateur dancers.

**DESIGN AND SETTING:**

Observational cross-sectional study; Curitiba, PR, Brazil.

**METHODS:**

Data on 150 professional and non-professional practitioners of ballet, jazz and street dance were collected through specific questionnaires: Brief Pain Inventory-Short Form (BPI-SF), Eating Attitudes Test-26 (EAT-26), Bulimic Investigatory Test Edinburgh (BITE) and State-Trait Anxiety Inventory-T-6 (STAI-T-6).

**RESULTS:**

Pain was observed in 58.6% of the sample, equally between professionals and amateurs (P = 0.19). Ballet dancers had more lower-limb pain than the other groups (P = 0.05). EAT-26 showed a tendency towards more eating disorders among the amateurs (P = 0.06). Higher risk of eating disorders was found among ballet dancers (P = 0.004) and jazz practitioners (P = 0.02) than among street dancers. Amateurs had more symptoms on the BITE scale (P < 0.0001), more pain (P = 0.002) and higher anxiety (P < 0.0001). Eating disorders were more common among females (P = 0.01) and singles (P = 0.02). Professionals were more satisfied with their own body image than amateurs (P < 0.001).

**CONCLUSIONS:**

Pain symptoms were found in almost half of the sample, equally among professionals and amateurs as well as between the three dance styles. Female and singles had more eating disorders. Those with eating disorders had higher levels of pain and anxiety.

## RESUMO

**CONTEXTO E OBJETIVO:**

Buscar a perfeição pode causar ansiedade e levar bailarinos a ultrapassar seus limites físicos. O objetivo foi avaliar a prevalência de sintomas dolorosos e distúrbios alimentares entre bailarinos profissionais e amadores.

**TIPO E LOCAL:**

Estudo transversal observacional; Curitiba, PR, Brasil.

**MÉTODOS:**

Dados de 150 praticantes profissionais e não profissionais de *ballet*, *jazz* e *street dance* foram coletados por meio de questionários específicos: Brief Pain Inventory-Short Form (BPI-SF), Eating Attitudes Test-26 (EAT-26), Bulimic Investigatory Test Edinburgh (BITE) e State-Trait Anxiety Inventory-T-6 (STAI-T-6).

**RESULTADOS:**

Encontrou-se dor em 58,6% da amostra, igualmente entre profissionais e amadores (P = 0,19). Praticantes de *ballet* tinham mais dor em membros inferiores que os demais (P = 0,05). No EAT- 26, encontrou-se uma tendência para mais transtornos alimentares entre os amadores (P = 0,06). Alto risco para transtornos alimentares apareceu naqueles que praticavam o *ballet* (P = 0,004) e *jazz* (P = 0,02) mais do que *street dance*; amadores tinham mais sintomas no BITE (P < 0,0001), mais dor (P = 0,002) e ansiedade (P < 0,0001). Transtornos alimentares foram mais comuns em mulheres (P = 0,01) e solteiros (P = 0,02). Bailarinos profissionais estavam mais satisfeitos com sua imagem corporal do que amadores (P < 0,001).

**CONCLUSÕES:**

Encontrou-se sintomatologia dolorosa em quase metade da amostra, tanto em bailarinos profissionais como amadores, bem como nos três estilos de dança. Mulheres e pessoas solteiras tiveram mais transtornos alimentares. Aqueles com distúrbios alimentares tinham níveis mais elevados de dor e ansiedade.

## INTRODUCTION

Dance is one of the most primitive forms of artistic expression. Dancers use their own bodies to communicate ideas and feelings. Therefore, it is natural that they seek the ideal physical shape, in order to practice their art with perfection. This search often leads them to subject their bodies to strenuous exercise and restrictive diets that turn them into a risk group for eating disorders and musculoskeletal injuries.^1^^,^^2^^,^^3^^,^^4^^,^^5^

Dancing ballet is demanding. It requires athletic ability and aerobic endurance, muscular strength, flexibility, good joint stability and neuromuscular coordination. The movements are complex and may involve a great range of motion. Therefore, mobility, static and dynamic strength, stability and equilibrium are necessary for extended periods. In addition, jumps may cause major impact on the feet. For these reasons, ballet dancers frequently overload bone structures, muscles and periarticular ligaments.^3^^,^^6^

Jazz and street dance require agility, since these forms explore the dynamics of body movements. Such movements need to be sharp to allow fast and accurate changes in body position, which requires neuromotor control, strength, speed, coordination, flexibility and balance.^7^ Thus, strain injuries are also common in this group of dancers.^8^

According to Arabia et al.^3^ the highest incidence of musculoskeletal injuries appears among dancers between 8 and 16 years of age. The risk factors include poor postural behavior, anatomical anomalies, improper dance technique, little training and muscle imbalance. In addition, practicing on oblique, slippery and unstable floors, as well as alternating scenarios between high brightness and darkness, and use of fog or smoke on stage, contribute to injuries. Very low body weight in association with low bone mass may favor stress fractures.^3^^,^^4^

In a study by Azevedo et al.^9^ that included 100 dancers, the risk factors for injury were: physical and general fatigue (mentioned by 53%); and unsuitable floors for dance practice and continuous repetition of demanding choreographic movements (both mentioned by 43.9%). The dancers indicated that the following items might prevent musculoskeletal problems: proper floors on stages and studios; and health professionals integrated into the dance company.

Early diagnosis and correct treatment of musculoskeletal injury are important because chronic pain has negative effects on individuals’ physical and mental state alike, thereby impairing quality of life.^10^

Dancers are also at risk of developing eating disorders because of the stringent requirements of physical performance and body esthetics. The onset of an eating disorder is mostly influenced by body image, more than by the individual’s weight.^5^ According to Hsu et al.,^11^ the variables involved in misperception of body image are gender, psychiatric disorders, age, maternal attitude (mainly among children and adolescents) and psychiatric disorders. Teenagers are the group most affected within this context, possibly because of their changes in lifestyle and self-confidence as they mature into adulthood. Misperception of weight has been correlated with lower satisfaction with life and poor self-rated health. Likewise, an association between perfectionism and eating disorder symptoms has been found.

Insufficient daily calorie intake over a long period generates loss of muscle mass, menstrual irregularities and inadequate bone mineralization.^4^ Eating disorders in dancers are known to be common, but the exact rates remain to be clarified. They may differ according to the region studied given that dietary habits may be influenced by cultural, social and economic factors.^12^

Evaluating painful symptoms and eating disorders among dancers allows researchers to find out about their routines, the environment within which they live and work and their quality of life. Such analyses can provide knowledge about the amount of physical and emotional stress to which these individuals are subjected and may contribute towards better understanding, recognition, diagnosis and treatment of pain and eating disorders in this population. Furthermore, very little is known about amateur dancers, since most studies have been conducted among professionals. Within this context, the present study aimed to investigate the prevalence of pain and eating disorders among dancers. It also aimed to detect whether there are differences in these issues among dancers practicing different dance styles and between professionals and amateurs.

## OBJECTIVE

The present study aimed to ascertain the prevalence of pain and eating disorders among dancer in one institution. It also aimed to determine whether any differences in these two issues exist among dancers practicing different dance styles and between professionals and amateurs, and to compare such characteristics between dancers presenting high and low risk of eating disorders.

## METHODS

This study was approved by the local Research Ethics Committee, and the participants or their legal guardians accepted the informed consent statement. This was an analytical cross-sectional study on a completely random sample formed by professional and amateur dancers aged 13 years and over. It was conducted through online virtual or paper versions of questionnaires applied at two dance schools in Curitiba, Paraná, Brazil. The sample comprised 150 dancers who were practicing ballet, jazz and street dance. All participants answered the questionnaires anonymously. Some dancers (n = 52) were invited to answer printed questionnaires that were distributed according to their order of arrival at classes and were later on collected by the school clerk. The online questionnaires (n = 98) were answered through an online form that had been specially designed for this study and advertised through social media (the dancers’ Facebook and internet groups). Only four of the invited dancers refused to participate: three of them claimed that they did not have time; the other one did not give any reason.

The participants of this study were asked to fill out the Brief Pain Inventory-Short Form (BPI-SF).^8^ The BPI-SF contains questions that evaluate pain, its intensity and its interference in normal activities. It is scored on a scale from 0 to 10 points, where 0 means the lowest and 10 the highest pain or interference in daily activities, mood, sleep and relationships. It also analyzes the usage of pain medication.

 The Eating Attitudes Test-26 (EAT-26)^13^^,^^14^^,^^15^ and Bulimic Investigatory Test Edinburgh (BITE)^13^ were used to assess eating disorders. EAT-26 contains 26 questions concerning eating attitudes, and its total score can range from 0 to 78 points. Scores ≥ 20 mean high risks of an eating disorder. This instrument investigates food selection, knowledge about calorie intake, perceptions about how other people evaluate the subject’s dietary habits, vomiting and its frequency, and self-control regarding the amount and frequency of food intake.

The BITE questionnaire has two scales: one on symptoms and the other on their severity. This questionnaire investigates the frequency of meals, food intake, fasting habits and body self-image. The symptom scale goes from 0 to 30 points and subjects with scores ≥ 20 are regarded as having a high risk of eating compulsion or bulimia; scores from 10 to 19, intermediate risk; and < 10, low risk. On the other hand, the severity scale ranges from 0 to 39 points, such that scores ≥ 5 express the need to investigate eating disorders and scores ≥ 10 reveal the need to treat eating disorders or bulimia.

The State-Trait Anxiety Inventory-6 (STAI-T-6)^16^ assesses self-report items pertaining to anxiety. Its scores can range from 6 to 24 points and higher scores indicate higher levels of anxiety.

All the questionnaires used had previously been translated and validated for the Portuguese language.^8^^,^^13^^,^^14^^,^^15^^,^^16^

For the statistical analysis, the data were gathered into contingency and frequency tables. The distribution was judged by means of the Kolmogorov-Smirnov test and central trend measurements were expressed as means and standard deviations in normal samples and as medians and interquartile ranges (IQR) in non-Gaussian samples. For analyses on nominal data, we used Fisher’s test and chi-square tests. For analyses on associations of numerical data, we used the Kruskal-Wallis test (for sets of three samples) and the Mann-Whitney test (for sets of two samples). The significance level of 5% was used and calculations were done with the aid of the GraphPad Prism software, version 5.0.

## RESULTS

### a) Descriptive analysis on the sample

Between May and August 2015, 150 dancers were interviewed: 48 professionals (members of dance companies) (32%) and 102 amateurs (68%). Regarding dance styles, 56% reported practicing classical ballet, 62.6% jazz dance and 24.6% street dance.

Comparisons of the epidemiological data and habitual dance styles between the professional and non-professional dancers are presented in Table 1. It can be seen that the professional dancers were practicing for more hours/week and that the proportion of male subjects was higher among the professionals than among the amateurs.

**Table 1.**
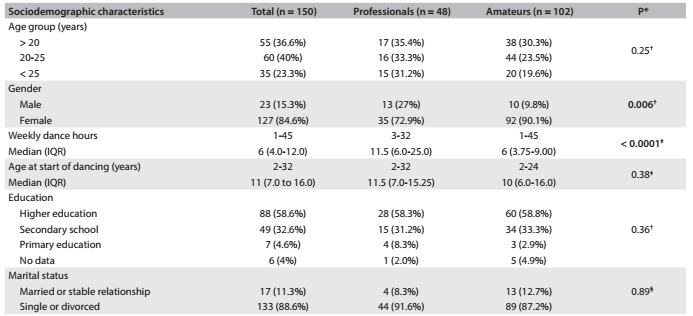
Comparison of epidemiological data and dancing habits between professional and non-professional dancers

*Refers to professionals versus amateurs; ^†^Chi-square test; ^‡^Mann-Whitney test; ^§^Fisher test. IQR = interquartile range.

### b) Pain evaluation

In the pain analysis using the BPI-SF, 87/150 (58%) of the participants declared that they had some kind of musculoskeletal pain (60.4% of the professionals versus 56.8% of the amateur group; with P = 0.19). In 82/150 (53.3%), the pain was mild, in 9/150 (6%) moderate and in 1/150 (0.6%) severe.

**Figure 1.**
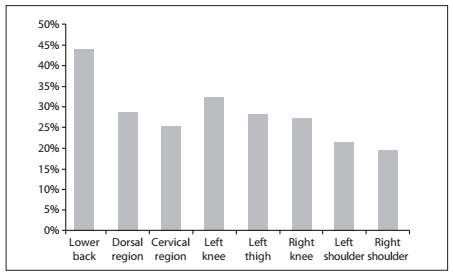
Most common sites affected by musculoskeletal pain among dancers (n = 150).

Figure 1 indicates the locations that were most affected. It can be seen that the highest rates of pain were in the spinal region and lower limbs. The most affected places were the spine (lower back: 44%; cervical spine: 25.3%; middle back: 28.6%), lower limbs (left knee: 32%; right knee: 27.3%; left thigh: 28%) and shoulder (right shoulder: 19.3%; left shoulder: 21.3%). Comparison of the pain regions showed that there was no statistical difference between professional and amateur dancers; however, classical ballet practitioners had more pain in the lower limb than did jazz and street dance practitioners (P = 0.05).

Regarding pain treatment methods (use of analgesics, anti-inflammatory drugs hormonal therapy or physical methods), professional dancers used more physical methods (such as physiotherapy, acupuncture, massage, etc.) than did the amateurs (P = 0.05).

Table 2 shows a comparison of the impact of pain on daily activities between the professional and non-professional dancers. In comparing the pain component by means of BPI-SF between the three dance forms, no significant difference (P = 0.65) was found.

**Table 2.**
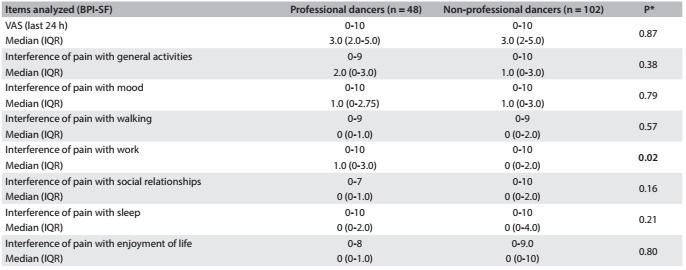
Comparison of pain according to BPI-SF (Brief Pain Inventory-Short Form) between professional dancers and amateurs

*Mann-Whitney test; VAS = visual analogue scale; IQR = interquartile range.

### c) Evaluation of dietary habits

Analysis on dietary habits using the EAT-26 questionnaire showed that 52/150 (34.6%) of all the participants had results consistent with a high risk of eating disorders and 98/150 (65.3%) had a low risk. This comparison of the sample with high and low risks of eating disorders according to EAT-26 is in Table 3. It shows that females and ballet dancers had higher risk of presenting eating disorders, while single or divorced dancers and jazz practitioners had less risk.

**Table 3.**
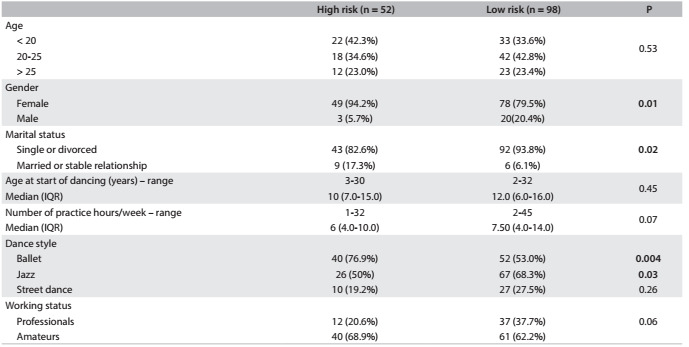
Comparison of dancers with high and low risk of eating disorders according to the EAT-26 questionnaire (Eating Attitudes Test-26)

Tests: Fisher; Chi-square; Mann Whitney; STAI-T-6 = State-Trait Anxiety Inventory T-6.

**Figure 2.**
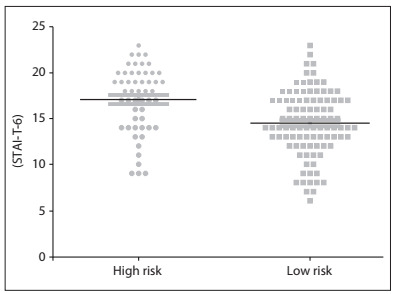
Comparison of anxiety scale (STAI-T-6) results among dancers with high and low risk of eating disorders (P < 0.0001; Mann-Whitney).

Figure 2 shows a comparison of the anxiety scale (STAI-T-6) results between subjects with high and low risk of eating disorders.

Interpretation of the results from the BITE scale for eating compulsion or bulimia showed that high scores were found in 14.6% of the sample and average scores in 43.3%. Severity domain analysis on this scale showed that 15.3% of the dancers had clinically significant values and 6% had very high scores. Professionals and amateurs had the same risk of bulimia (P = 0.39) and the frequency of meals (breakfast, lunch, dinner and snacks) was the same in both groups (all meals with P = non-significant).

There was no difference in body mass index between the professional and non-professional dancers (P = 0.28), and also no differences among the three dance styles studied (P = 0.33). However, investigation of personal views of current weight versus ideal weight showed that the amateurs were less satisfied with their own body image than were the professionals (P < 0.001).

## DISCUSSION

Our results showed that more than half of the sample analyzed had some kind of musculoskeletal pain and that dance style did not affect dancers’ perceptions regarding appearance. Moreover, as expected, pain among the professional dancers played an important role regarding interference with their work. However, the number of hours a week dance practice was highly variable in both professional and amateur groups. Eating disorders were observed in almost one third of the study sample, mainly occurring among females, ballet practitioners and those with high levels of anxiety. Interestingly, eating disorders were less common among singles and jazz practitioners. It was also observed that almost 20% of the dancers presented a risk of bulimia and that this risk was equally distributed between professionals and amateurs. Finally, it could be seen that the amateur dancers showed great dissatisfaction with their own body image.

Physical activity reduces pain levels^10^ and, moreover, the act of dancing relieves tension and day-to-day stress. However, despite its benefits for physical fitness and pleasure, dancing requires hours of training and it takes years to improve performance, which may generate situations of stress and anxiety. The search for perfection may lead to excessive physical activity, which possibly leads to musculoskeletal injuries. This cycle ultimately allows the act of dancing to be considered to be an important occupational disease.^2^^,^^17^ Furthermore, as part of the physically active population, dancers are prone to accidents.^2^ Almost 100% of retired dancers who belonged to the ballet companies American Ballet and New York City Ballet were found to have had at least one injury during their careers.^18^

Pain cannot be objectively assessed by tests, and its evaluation depends on the patient’s report.^10^ This serves to emphasize the importance of applying instruments that can quantify perceived pain and its repercussions on dancers’ daily lives and work, such as the BPI-SF questionnaire used here.

As stated earlier, more than half of the dancers had some sort of musculoskeletal pain and the most common injury sites were the lower spine, followed by the middle back and cervical regions and lower limbs. These results agree with those of the study by Dore and Guerra,^8^ on 141 professional ballet dancers who showed similar affected regions (low back, knees, neck and left hip/thigh). It can be stated that the high prevalence of pain in the low back and legs is due to the classical dance position of extreme external rotation of the lower limbs, which leads to hyperlordosis, and also to the weight supported on the toes through tip and half-tip positions. Weak abdominal muscles and tight thoracolumbar fascia may favor this, thus increasing the chance of injury from a curved back and arabesques. Accordingly, ballet dancers were the ones who had most pain in the lower limbs. However, in relation to the other regions, including the lower back, there were significant differences among the dance styles studied. This suggests that further studies should be conducted in order to evaluate the causes of pain in these regions, since the positions and repetitions used in different types of dance diverge from one style to another. We believe that jazz dancers reported more pain in the lower limbs than did street dancers because of the high similarities between jazz positions and those performed in classical ballet.

Regarding the intensity of pain, although reports of mild pain prevailed in the present study (53.3%), Dore and Guerra^8^ found high levels of pain in 70.2% of the dancers. They also demonstrated the influence of pain on work activities. In our comparison between professional and amateur dancers, we found a significant difference in the interference with work produced by pain (P = 0.02), such that this relationship was closer among the professionals. We therefore reiterate the above authors’ assertion that painful symptoms interfere significantly in the work activities of dancers, especially among professionals, since they use their bodies as a work tool. Thus, it is necessary to implement or enhance measures to prevent injury and improve the treatment of existing lesions, and to place dancers under constant monitoring by healthcare professionals. The presence of medical staff in dance companies helps to reduce injuries and the number of days off work due to illness, and saves financial resources among dancers.^4^ According to the literature, the most successful model is built up through a multidisciplinary team that includes dance teachers, doctors, physiotherapists, massage therapists, Pilates instructors, sports psychologists and sports nutritionists.^18^

Eating disorders are entities of multifactorial origin with higher occurrence rates among teenagers and young women, and with significant prevalence in the general population. It is also important to point out that late diagnosis, due to denial of the situation, ultimately leads to health impairment.^19^ In the present study, almost one third of the dancers (34.6%) were at risk of eating disorders, half of them with high scores. We also found that younger people, females, single individuals and classical ballet practitioners were more vulnerable to this condition. Guimarães et al.^20^ also found that a high proportion of classical ballet dancers had eating disorders and showed dissatisfaction with body image. In the present study, the BMI of dancers of all styles was found to be within the normal range. Despite this, 54.1% of the professional dancers and 96% of the amateur dancers classified themselves as being overweight. This shows that acknowledgment of body image seems to be disturbed mainly in the amateur group. Individuals with eating disorders, and specifically bulimia, usually place extreme value on their body shape and weight. They have erroneous physical perceptions, difficulty in identifying emotions, low self-esteem, a low threshold for frustration, impaired impulse control and high levels of anxiety.^21^ The requirement for dancers to maintain an extremely low weight is closely linked to the athletes’ triad (eating disorders, amenorrhea and osteoporosis).^3^^,^^4^

Unfortunately, the lack of references involving jazz and street dance styles hinders comparisons of the present data with other samples, which thus suggests that there is a need for more studies within this context. The same applies to the group of amateur dancers, which was the larger group in the present study and also showed a great degree of musculoskeletal pain and eating behavior disorders.

Finally, this study provides some knowledge about the lives of dancers and shows that this group needs support with regard to both of the situations studied: prevention of musculoskeletal pain and counseling to improve dietary habits. This study also highlights that this guidance is needed not only by professionals but also by amateurs, who are at similar risk of both musculoskeletal lesions and eating disorders. However, lastly, this was only a small cross-sectional study. For a better panorama to be provided, this study needs to be replicated with larger samples that are evaluated by a multidisciplinary team that should include psychologists in order to better understand the relationship between the problems observed and their causes.

## CONCLUSIONS

Painful symptoms were commonly observed and found at similar rates among professional and amateur dancers, and also among dancers practicing the three styles that were studied. The back and lower limbs were the regions most affected by pain, which had greater interference with work activities among professional dancers.

Almost one third of the dancers were at risk of developing eating disorders, which were more commonly seen among young and single females. Regarding self-awareness of the body, amateur dancers had worse body image perceptions than the professionals. Dancers presenting a high risk of eating disorders had higher levels of pain and anxiety than those at low risk.
